# Exosomal let‐7c‐5p From AMI Patient Plasma Modulates Neuronal PANoptosis via ZBP1 In Vitro

**DOI:** 10.1002/cbf.70318

**Published:** 2026-10-08

**Authors:** Zhiyong Zhang, Shaohan Zhang, Yuzhi Shao, Zhiguang Wang, Lei Yan, Xinyu Wang, Wei Li, Kerui Gong, Gang Fu, Jie Yan, Wendian Yang, Guo Shao

**Affiliations:** ^1^ Center for Translational Medicine, Clinical lnstitute of Shantou University Medical College The Third People's Hospital of Longgang District Shenzhen Shenzhen China; ^2^ Inner Mongolia Key Laboratory of Hypoxic Translational Medicine Baotou Medical College Baotou China; ^3^ Beijing Key Laboratory of Hypoxic Conditioning Translational Medicine Xuanwu Hospital of Capital Medical University Beijing China; ^4^ Traditional Chinese Medicine Department Tianjin University of Traditional Chinese Medicine Tianjin China; ^5^ School of Life Science Guangzhou University Guangzhou China; ^6^ School of Basic Medicine Guangdong Pharmaceutical University Guangzhou China; ^7^ Department of Neurological Surgery University of California San Francisco San Francisco California USA; ^8^ ICU, The Second People's Hospital of Longgang District Shenzhen China; ^9^ Joint Laboratory of South China Hospital Affiliated to Shenzhen University and Third People's Hospital of Longgang District Shenzhen University Shenzhen China

**Keywords:** acute myocardial infarction, brain injury, exosome, let‐7c‐5p, PANoptosis

## Abstract

Acute myocardial infarction (AMI) significantly elevates the risk of ischemic stroke (IS), yet the molecular mechanisms linking cardiac and cerebral injury remain unclear. Emerging evidence highlights the role of circulating exosomes in mediating inter‐organ communication via microRNAs (miRNAs). In this study, plasma exosomes from AMI patients were isolated at three time points (before, 2 hours after, and 24 hours after reperfusion therapy (RT)), and the exosomal let‐7c‐5p level was measured. The effects of plasma exosomes on neuronal SH‐SY5Y cells under normoxic and oxygen‐glucose deprivation (OGD) conditions were evaluated by assessing cell viability and the Z‐DNA binding protein 1 (ZBP1)/PANoptosis pathway using CCK‐8 assay and western blot analysis. let‐7c‐5p was overexpressed to clarify its role in the ZBP1/PANoptosis pathway. Exosomal let‐7c‐5p from AMI patients showed differences at three time points. Exosomes collected before reperfusion therapy induced the most PANoptosis under normal conditions, whereas those collected 2 hours post‐reperfusion therapy had the greatest effect under OGD. These effects correlated with dynamic changes in exosomal let‐7c‐5p levels and the expression of ZBP1. Let‐7c‐5p overexpression reduced ZBP1 and PANoptotic markers, suggesting a neuroprotective role under ischemic stress. Our study suggested that the exosomal let‐7c‐5p/ZBP1/PANoptosis axis may contribute to AMI‐related brain injury, offering potential therapeutic targets for intervention.

## Significance Statement

This study explored how acute myocardial infarction may influence the brain through plasma exosomes. Exosomal microRNA from patients with acute myocardial infarction was investigated. Plasma exosomal let‐7c‐5p changed over time after reperfusion therapy. These exosomes were found to be taken up by neuronal cells and regulate a key cell death pathway (ZBP1‐mediated PANoptosis). Importantly, upregulation of let‐7c‐5p reduced harmful cell death signals and improved neuronal survival under ischemic conditions. These findings revealed that exosomal miRNAs may play an important role in cardio‐cerebral communication following cardiac events and subsequent brain injury, and suggested the exosomal let‐7c‐5p/ZBP1 pathway as a promising target for preventing or treating post‐infarction neurological complications.

## 1 Introduction

Stroke and myocardial infarction (MI) are the leading causes of death worldwide, according to the World Health Organization [1]. Acute ST‐elevation myocardial infarction (STEMI) and acute ischemic stroke (AIS) share multiple pathophysiological features, highlighting a close and clinically significant heart–brain interaction within cardiovascular diseases [2]. Witt et al. found that stroke risk rose 44‐fold within 30 days after MI, dropped to 3.1‐fold between 31 days and 1 year, and stabilized to near‐normal levels (1.6‐fold) after 3–4 years [3]. Merkler et al. showed that the risk of ischemic stroke was highest within the first 4 weeks post‐myocardial infarction discharge, remained elevated through weeks 5–12 [4]. Therefore, the ischemic processes associated with myocardial infarction may influence the incidence of stroke due to their specific characteristics.

It has been reported that certain extracellular vesicle (EV) proteins and microRNAs (miRNAs) play roles in both myocardial infarction and stroke [1]. Exosomes are extracellular vesicles ranging from 40 to 200 nm in diameter, originating from diverse sources and containing various small molecules that facilitate intercellular signaling [5]. Research has demonstrated that circulating exosomal miRNAs are implicated in cardiovascular diseases and various other pathological processes, exhibiting anti‐inflammatory, anti‐apoptotic, and angiogenesis‐promoting properties that may enhance cardiac repair and influence the function of other organs [6]. Due to their widespread presence in plasma, exosomal miRNAs may serve as a crucial link between the heart and the brain.

MiRNA Let‐7 stands out both for its abundance and its high degree of conservation among miRNAs [7]. Let‐7 is highly expressed in the cerebral and cardiovascular systems, and aberrant expression of let‐7 family members has been implicated in vascular diseases such as stroke and myocardial infarction [8]. Let‐7c‐5p alleviates post‐stroke injury by suppressing caspase‐3 mediated microglial activation or by other mechanisms [9, 10]. Exosomal let‐7c‐5p can be used as a promising biomarker for the diagnosis of stable coronary artery disease [11]. This positions exosomal let‐7c‐5p as a plausible molecular nexus linking the pathophysiological crosstalk between the heart and the brain, potentially via the modulation of shared cell death mechanisms.

Regulated cell death is a central pathological feature shared by both myocardial infarction and ischemic stroke, particularly in the context of ischemia/reperfusion (I/R) injury. PANoptosis represents a distinct form of regulated cell death that combines elements of pyroptosis, apoptosis, and necroptosis into a unified pathway [12]. Studies have shown that pyroptosis, apoptosis, and necroptosis are simultaneously activated and integrated. This process is coordinated by the PANoptosome, a large heteromultimeric complex that acts as a master regulator by recruiting molecular components from all three death pathways [13]. Z‐DNA binding protein 1 (ZBP1) is a cytosolic sensor of Z‐form nucleic acids (Z‐NA) that promotes the formation of the ZBP1‐PANoptosome, triggering GSDMD‐dependent pyroptosis, caspase‐3 mediated apoptosis and MLKL driven necroptosis [14]. ZBP1 is upregulated in the peri‐infarct brain region following ischemic stroke, and its downregulation may help alleviate stroke induced pathological damage [15, 16]. PANoptosis is triggered by diverse intra‐ and extracellular signals that engage distinct pathways and molecular mechanisms. Some studies have indicated that PANoptosis can be triggered by I/R [17, 18]. The inhibition of PANoptosis can promote neurofunctional recovery after middle cerebral artery occlusion/reperfusion (MCAO/R) in rats and oxygen‐glucose deprivation/reoxygenation (OGD/R) in cultured neuronal cells [19, 20]. Therefore, this study aimed to investigate the mechanisms linking acute myocardial infarction (AMI) and ischemic stroke and to evaluate the potential effects of AMI reperfusion therapy on ischemic stroke via the ZBP1/PANoptosis pathway in vitro.

## 2 Materials and Methods

### 2.1 Subject Population

Six healthy male volunteers (aged 50–60 years, height 170–180 cm, weight 60–75 kg) were recruited for this study. All samples were collected in the early morning while participants were awake and fasting. Two milliliters of venous blood was drawn from each volunteer as a control sample. Six male patients (aged 50–60 years, height 170–180 cm, weight 60–75 kg) diagnosed with AMI at The Third People's Hospital of Longgang District, Shenzhen, China, between October 2024 and November 2024, were selected for coronary reperfusion therapy. From each patient, 2 mL of venous blood was collected at three time points: before reperfusion therapy, 2 h after reperfusion therapy, and 24 h after reperfusion therapy. These time points were selected to capture the baseline ischemic state, the early reperfusion phase, and a later stabilized phase [21]. This study was approved by the Ethics Committee of The Third People's Hospital of Longgang District Shenzhen (KY‐KT‐20240024). All participants signed informed consent before enrollment.

### 2.2 Isolation and Characterizations of Exosomes

Exosomes were extracted from blood with Hi‐Efficiency Exosome Precipitation Reagent (Invent Biotechnologies, MN, USA). The collected blood was centrifuged at 1000 × g for 10 min at 4°C to obtain plasma, and the plasma was uniformly mixed with phosphate‐buffered saline (PBS) at a ratio of 1:2. Subsequently, the samples were processed in accordance with the manufacturer's protocol. Exosome quantification was performed as previously described by Luhtala and Hunter [22]. Exosomes were identified by western blot and transmission electron microscopy, as detailed by Grigor'eva et al [23]. Then, the exosomes were stored at −80°C.

### 2.3 Bioinformatics Analytics and Prediction

Next‐generation sequencing (NGS) analysis revealed differentially expressed microRNAs (miRNAs). The miRDB.org website (https://mirdb.org/) was used to identify miRNAs related to ZBP1. Bielefeld Bioinformatics Service software (https://bibiserv.cebitec.uni-bielefeld.de/) was used to validate them.

### 2.4 Cell Culture, Exosome Incubation, and OGD/R Treatment

Human neuroblastoma SH‐SY5Y cells were obtained from the Chinese Academy of Sciences Cell Bank (SCSP‐5012; ATCC CRL‐2266) and authenticated by STR profiling, showing 100% match with the Cellosaurus database (CVCL_0019). SH‐SY5Y cells were cultured in DMEM/F12 (Servicebio, Wuhan, China) supplemented with 10% fetal bovine serum (FBS) (Gibco, Paisley, UK) and 1% penicillin/streptomycin. Exosomes from healthy volunteers and patients plasma were incubated with the cells (approximately 35 µg exosomes/mL of medium) for 24 h prior to subsequent experiments. The experimental groups were as follows: Blank (normal culture), C (exosomes from healthy volunteers), B (AMI exosomes before reperfusion therapy), 2 h (AMI exosomes 2 h after reperfusion therapy), and 24 h (AMI exosomes 24 h after reperfusion therapy). After incubation, cells were either cultured for an additional 24 h or subjected to OGD/R treatment. OGD/R treatment was performed as follows: SH‐SY5Y cells were incubated in glucose free DMEM under hypoxic conditions (5% CO_2_, 95% N_2_) for 6 h, followed by reoxygenation in standard medium for 18 h.

### 2.5 Exosomal Tracking Experiment

The lipophilic tracer DiI (BBI Life Sciences Co. Ltd., Shanghai, China) was dissolved in Dimethyl sulfoxide (DMSO) (Sangon Biotech, Shanghai, China). Exosomes derived from the control group and the AMI patient group were incubated with 5 μM DiI at 37°C for 20 min. Excess dye was removed by washing the samples three times with PBS, then centrifuging. The DiI labeled exosomes were then incubated with SH‐SY5Y neuronal cells for 24 h. After incubation, the cells were rinsed with PBS and fixed with 70% pre‐cooled ethanol for 5 min. Following fixation, the cells were rinsed twice with PBS and stained with 4',6‐diamidino‐2‐phenylindole dihydrochloride (DAPI) (Sangon Biotech, Shanghai, China). The cells were visualized using a laser confocal microscope (LSM 900, ZEISS, Germany).

### 2.6 Cell Viability

Cell viability was determined using the CCK‐8 assay (Beyotime Institute of Biotechnology, Shanghai, China). According to the manufacturer's protocol, SH‐SY5Y cells were seeded into 96‐well plates at a density of 5 × 10^3^ cells/well and incubated at 37°C. The next day, cells in each well were washed with PBS and then cultured with 10 µL CCK‐8 reagent for 2 h at 37°C. Absorbance, a measure of cell viability, was measured at 450 nm using microplate reader.

### 2.7 Immunofluorescence

The cells were seeded into laser confocal glass bottom cell culture dish (Biosharp, Beijing, China). When the cell density reached approximately 70%, the medium was discarded, and the cells were rinsed with PBS. Pre‐cooled 70% ethanol was then added dropwise to fully cover the bottom of the dish for fixation (5 min), followed by two PBS rinses. After fixation, the cells were permeabilized with 1.5 M HCl for 1 h and rinsed twice with PBS. Blocking was performed using 5% BSA for 1 h. The primary antibody (diluted 1:200) was applied and incubated for 1 h at room temperature, followed by overnight incubation at 4°C. After rinsing, the secondary antibody (diluted 1:1000) was incubated at room temperature for 2 h in the dark. Nuclei were stained with DAPI at room temperature for 20 min, followed by a PBS rinse. Finally, laser confocal microscopy (LSM 900, ZEISS, Germany) was used to observe fluorescence and cellular status.

### 2.8 Lentivirus Transfection

SH‐SY5Y cells were counted and then cultured in 24‐well plates at a density of 1 × 10^5^ cells per well. When SH‐SY5Y cells reached 50% confluency, the let‐7c‐5p and control lentiviral vectors (Hambio Biotech, Shanghai, China) were added to the well plates to co‐culture with the cells (MOI = 20). After the vectors were transferred into the cells, stabilized cells were selected with 3 μg/mL puromycin dihydrochloride hydrate (Sangon Biotech, Shanghai, China).

### 2.9 RT‐qPCR Detection

After lysing exosomes and cells with Trizol (Invitrogen, Carlsbad, CA, USA), the resulting suspension was used to extract total RNA. MiRNA reverse transcription kits (TaKaRa, Beijing, China) were used for the first‐strand synthesis of miRNA. Moreover, cDNA was synthesized with a cDNA synthesis kit (TaKaRa, Beijing, China). The expression levels of let‐7c‐5p and ZBP1 mRNA were detected by RT–qPCR. U6 snRNA and β‐actin were used as internal references for let‐7c‐5p and ZBP1, respectively. All procedures were performed according to the manufacturer's instructions. Relative expression levels were then calculated using the 2^(−ΔΔCt) method. The PCR primers of let‐7c‐5p and ZBP1 were synthesized by Sangon Biotech (Shanghai, China). The synthesized primer sequences for let‐7c‐5p are: forward (F) 5'‐TGAGGTAGTAGGTTGTATGGTT–3', reverse (R) 5'‐TGAGGTAGTAGGTTGTATGGTT–3'. The primer sequences for ZBP1 are: forward (F) 5'‐AACATGCAGCTACAATTCCAGA–3', reverse (R) 5'‐AGTCTCGGTTCACATCTTTTGC–3'.

### 2.10 Western Blot

Cells were lysed and proteins extracted by radioimmunoprecipitation assay (RIPA) lysis buffer and protease inhibitors (Beyotime Institute of Biotechnology, Shanghai, China). Total protein concentration was determined by bicinchoninic acid (BCA) assay kit (MeilunBio, Dalian, China). The extracted proteins were subjected to sodium dodecyl sulfate‐polyacrylamide gel electrophoresis (SDS‐PAGE). The separated proteins were then transferred to a polyvinylidene difluoride (PVDF) membrane (Millipore, Jaffrey, NH, USA). The membranes were incubated with 5% skim milk blocking buffer for 1 h at room temperature. Then the membranes were incubated with ZBP1 (1:1500), Caspase‐8 (1:2000), NLRP3 (1:1000), RIP3 (1:2000), Caspase‐3 (1:2000), GSDMD (1:2000), GSDME (1:2000), and MLKL (1:1000) antibodies (all from Proteintech Group, Wuhan, China) at 4°C overnight. After being washed, the membranes were incubated with the corresponding HRP‐conjugated secondary antibodies (Sangon Biotech, Shanghai, China) for 1 h at room temperature. Protein bands were detected using an enhanced chemiluminescence (ECL) substrate (4 A Biotech, Suzhou, China) and visualized using an automated chemiluminescence imaging system (Tanon IVDT‐1, Guangzhou, China).

### 2.11 Statistical Analysis

The experimental data were expressed as the mean ± standard error of the mean (SEM). Statistical analyses were completed by one‐way analysis of variance (ANOVA) using SPSS 17.0 software. Moreover, the Tukey test was used to determine whether the difference between any two groups was significant. Statistical results were considered statistically significant when *p* < 0.05.

## 3 Results

### 3.1 Identification of Exosomes from the Plasma of AMI Patients

Exosomes were isolated from the plasma of patients with AMI using an exosome precipitation reagent [24]. To verify the identity of the isolated vesicles, their morphology was examined by transmission electron microscopy (TEM). TEM analysis revealed vesicles with cup‐shaped, round, or ellipsoidal morphology, consistent with the typical morphology of exosomes (Figure 1A). In addition, Western blot analysis confirmed the presence of established exosomal markers, including HSP70, TSG101, and CD63 (Figure 1B), verifying the successful isolation of exosomes.

**Figure 1:**
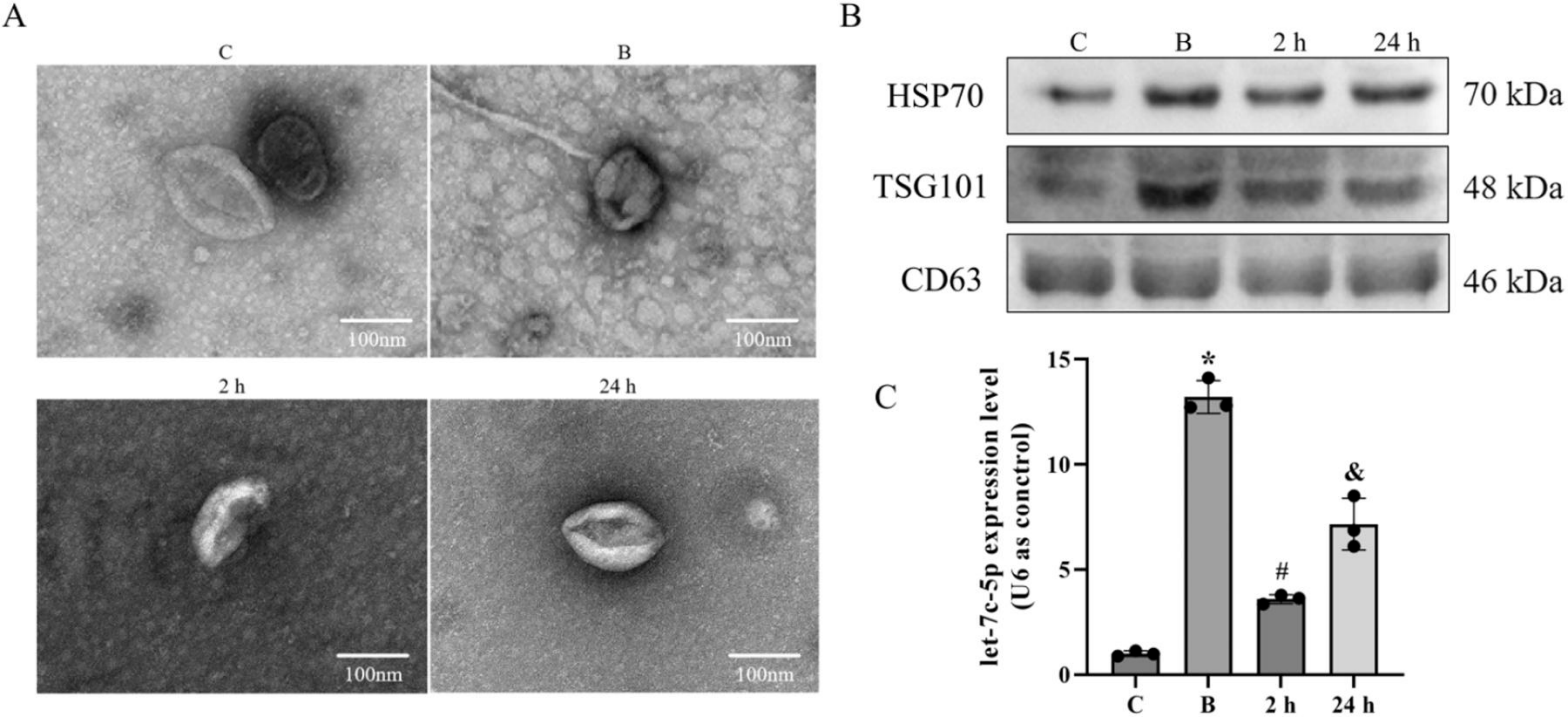
Identification of exosomes and exosomal let‐7c‐5p levels from AMI patients. (A) Transmission electron microscopy images of exosomes; (B) Western blot analysis confirming the expression of HSP70, TSG101, and CD63, markers of exosomes; (C) Expression of let‐7c‐5p in plasma exosomes from healthy individuals, preoperative patients, and patients at 2 h and 24 h after surgery. *p < 0.05 versus all other groups; ^#^ *p* < 0.05 versus C and 24 h groups; ^&^ *p* < 0.05 vs C. *n* = 3.

### 3.2 Bioinformatic Analysis of Plasma Exosomal microRNA and Measured Its let‐7c‐5p Level

Next‐generation sequencing (NGS) revealed differential expressions of exosomal miRNAs at three time points: before reperfusion and at 2 and 24 h post‐reperfusion therapy. Functional enrichment analyses based on the KEGG and GO databases indicated that the differentially expressed miRNAs were significantly associated with diverse biological pathways and molecular mechanisms. These pathways included vascular smooth muscle contraction and HIF‐1 mediated signaling, as well as functions related to signal transduction and transcriptional regulation (Supplementary Data, Figure 1).

As shown in Figure 1C, RT‐qPCR analysis revealed that let‐7c‐5p expression levels were significantly elevated in preoperative AMI‐derived exosomes compared with the control group and the 2‐ and 24‐h post‐reperfusion groups (*p* < 0.05). Notably, let‐7c‐5p expression was significantly reduced at 2 h post‐reperfusion compared with the control, preoperative, and 24‐h post‐reperfusion groups (*p* < 0.05). A partial recovery of let‐7c‐5p expression was observed at 24 h post‐reperfusion compared with the 2‐h post‐reperfusion group (*p* < 0.05).

### 3.3 Exosomes from AMI Patient Plasma Are Taken up by SH‐SY5Y Neuronal Cells and Modulate ZBP1 Expression and Panoptosis under Normal Conditions

SH‐SY5Y neuronal cells are capable of internalizing plasma‐derived exosomes. Confocal laser scanning microscopy revealed that SH‐SY5Y cells incubated with DiI‐labeled exosomes exhibited discrete orange‐red punctate fluorescence, indicating successful uptake of exosomes derived from both healthy volunteers and AMI patients (Figure 2A).

**Figure 2:**
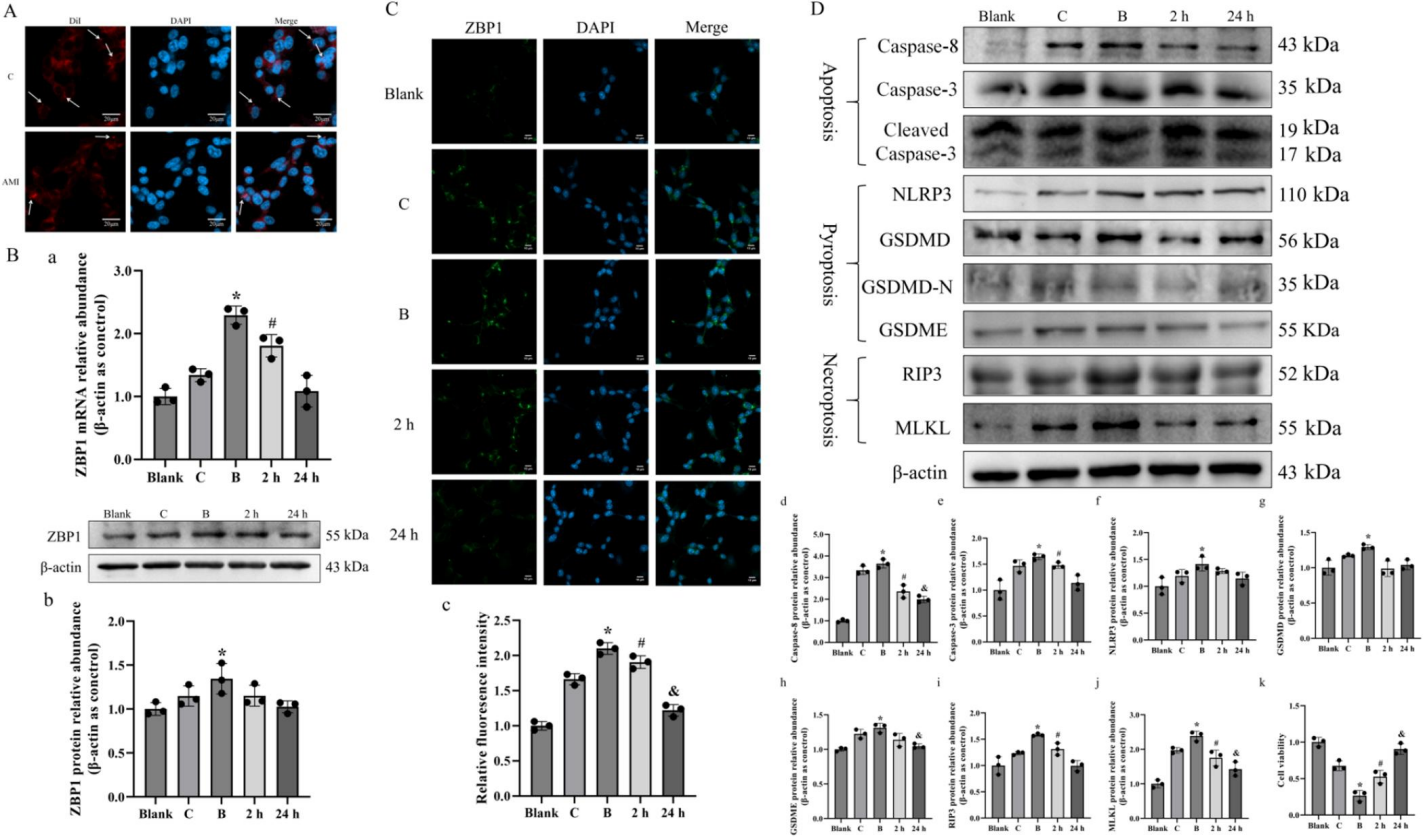
AMI plasma‐derived exosomes are taken up by SH‐SY5Y cells and affect ZBP1 expression and PANoptosis‐related protein levels. (A) Uptake of DiI‐labeled exosomes derived from healthy volunteers and AMI patients (red fluorescence) by SH‐SY5Y cells; (B) Real‐time PCR analysis of ZBP1 mRNA expression and western blot analysis of ZBP1 protein levels; (C) Immunofluorescence staining and quantification of ZBP1 relative fluorescence intensity; (D) Western blot analysis of PANoptosis‐related proteins: Caspase‐8, Caspase‐3, NLRP3, GSDMD, GSDME, RIP3, and MLKL; CCK‐8 assay measuring SH‐SY5Y cell viability following exosome treatment. *p < 0.05 compared with Blank (in all), C (in a, c, i, k), 2 h (in a, d, g, h, j, k) and 24 h (in a, b, c, d, e, g, h, i, j, k) groups; ^#^ *p* < 0.05 compared with Blank (in all), C (in a, c, d) and 24 h (in a, c, e, i, k) groups; ^&^ *p* < 0.05 compared with Blank (in c, d) and C (in c, d, h, j, k) groups. *n* = 3.

Plasma‐derived exosomes were collected at different time points (before, 2 h after, and 24 h after reperfusion therapy). SH‐SY5Y cells were then treated with these exosomes to evaluate the effects of AMI‐derived exosomes on cellular ZBP1 expression. ZBP1 expression levels were assessed by RT‐qPCR and western blot analysis. Both assays revealed that ZBP1 expression was highest in cells treated with preoperative exosomes. In contrast, ZBP1 expression gradually decreased following reperfusion therapy, with significantly lower levels observed at 24 h post‐reperfusion compared with preoperative levels (*p *< 0.05) (Figure 2B). Immunofluorescence analysis further supported these findings, showing significantly increased ZBP1 fluorescence intensity in cells treated with preoperative exosomes (*p *< 0.05) (Figure 2C).

As shown in Figure 2D, western blot analysis revealed significantly increased expression of PANoptosis‐related proteins in SH‐SY5Y cells treated with preoperative exosomes (*p* < 0.05), whereas lower expression levels were observed in the 2‐h and 24‐h post‐reperfusion groups. Consistently, CCK‐8 assays showed that cell viability was significantly reduced in the preoperative group compared with the post‐reperfusion groups (*p* < 0.05). Notably, cell viability partially recovered in the 2‐h and 24‐h groups.

### 3.4 Overexpression of let‐7c‐5p Inhibits ZBP1 Expression and PANoptosis in SH‐SY5Y Cells

Bioinformatic analysis predicted that ZBP1 is a putative target gene of let‐7c‐5p (Figure 3A). To validate this prediction, let‐7c‐5p was overexpressed in SH‐SY5Y cells via lentiviral transduction. Successful transduction was confirmed by the presence of strong green fluorescence in transduced cells (Figure 3B). Further RT‐qPCR analysis demonstrated that let‐7c‐5p expression was significantly increased in the overexpression group compared with the control group (*p* < 0.05), indicating effective overexpression of the target miRNA.

**Figure 3:**
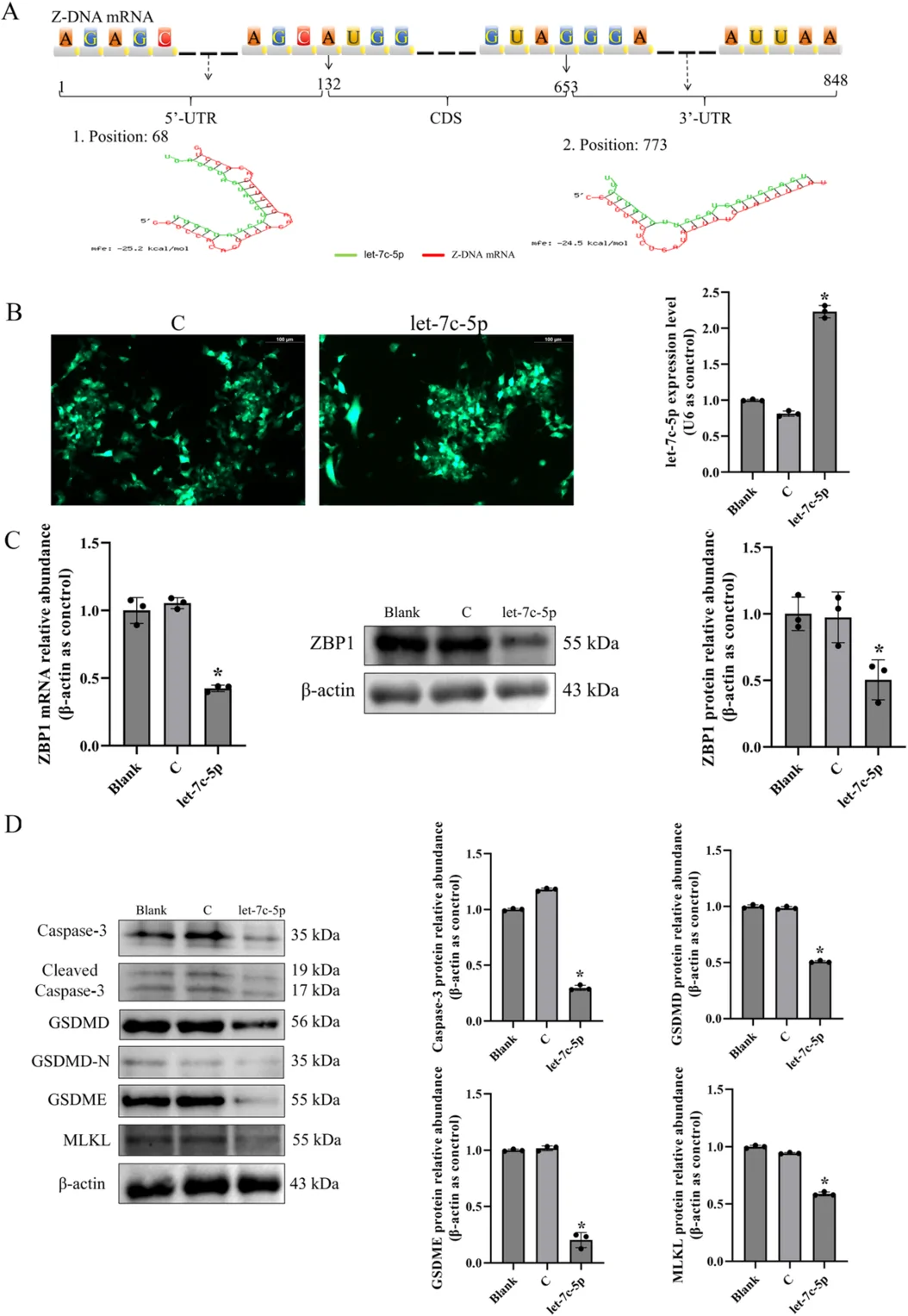
Predicted binding of let‐7c‐5p to ZBP1 mRNA and the effects of let‐7c‐5p overexpression on ZBP1 expression and PANoptosis‐related protein levels in SH‐SY5Y cells. (A) Bioinformatic prediction showing two potential binding sites of let‐7c‐5p in ZBP1 mRNA, located within the 3' UTR and 5' UTR. The predicted binding entropy values were 25.2 and 24.5 kcal/mol, respectively, suggesting direct regulation by let‐7c‐5p; (B) GFP fluorescence image of SH‐SY5Y cells 48 h after lentiviral transduction of let‐7c‐5p. RT‐qPCR analysis showing efficient overexpression of let‐7c‐5p; (C) RT‐qPCR analysis of ZBP1 mRNA expression and western blot analysis of ZBP1 protein levels; (D) Western blot analysis of PANoptosis‐related proteins, including Caspase‐3, GSDMD, GSDME, and MLKL. *p < 0.05 versus Blank and C groups. *n* = 3.

Consistently, both mRNA and protein expression levels of ZBP1 were significantly reduced following let‐7c‐5p overexpression compared with the control group (*p *< 0.05) (Figure 3C), supporting the notion that let‐7c‐5p negatively regulates ZBP1 expression.

To investigate the downstream effects of let‐7c‐5p mediated ZBP1 suppression on PANoptosis, the expression of proteins involved in PANoptosis execution, which are key effector proteins in apoptosis (Caspase‐3), pyroptosis (GSDMD, GSDME), and necroptosis (MLKL) was analyzed. The protein expression levels of Caspase‐3, GSDMD, GSDME, and MLKL were significantly reduced in the let‐7c‐5p overexpression group compared to the control group (*p *< 0.05) (Figure 3D). At the same time, no significant differences were observed between the blank and control groups (*p* < 0.05). These results paralleled the observed ZBP1 expression trends and suggest that overexpression of let‐7c‐5p attenuates PANoptosis in neuronal cells by downregulating ZBP1.

### 3.5 Exosomes From AMI Patient Plasma Modulate ZBP1 Expression and Panoptosis Under OGD/R

To further investigate the effects of exosomes under ischemic stress, SH‐SY5Y cells were subjected to oxygen‐glucose deprivation/reoxygenation (OGD/R) following exosome incubation. RT‐qPCR and western blot analyses confirmed that ZBP1 mRNA and protein levels were highest in the OGD‐Blank and OGD‐2 h groups and were significantly reduced in the OGD‐24 h group (*p* < 0.05) (Figure 4A). Immunofluorescence analysis revealed that ZBP1 expression was significantly elevated in the OGD‐Blank and OGD‐2 h groups compared with the other groups (*p* < 0.05). Meanwhile, exosomes collected at 24 h post‐reperfusion markedly reduced ZBP1 expression (*p *< 0.05) (Figure 4B).

**Figure 4:**
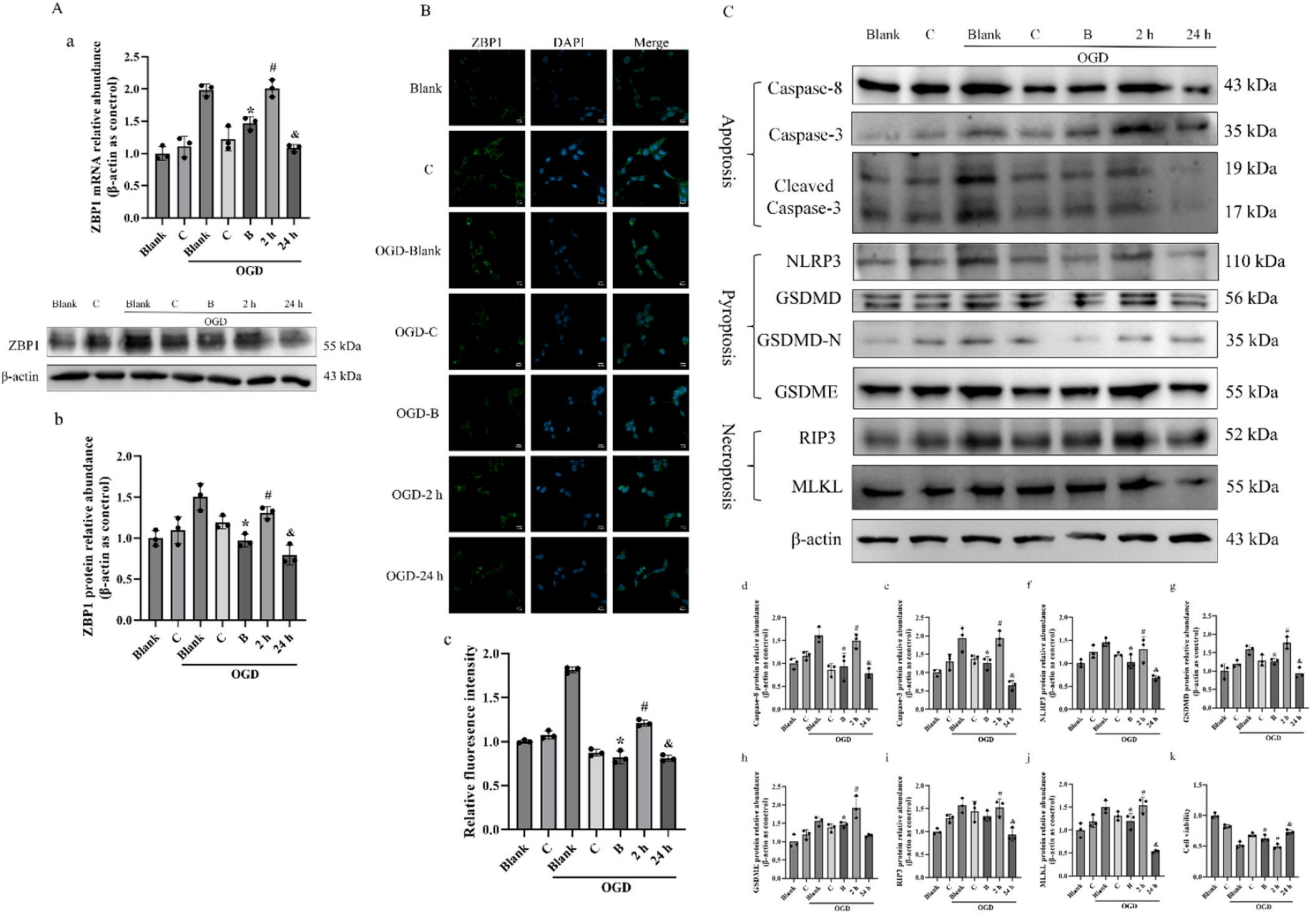
Effects of AMI plasma‐derived exosomes on ZBP1 expression and PANoptosis in SH‐SY5Y cells under OGD/R conditions. (A) Real‐time PCR analysis of ZBP1 mRNA expression and western blot analysis of ZBP1 protein levels; (B) Immunofluorescence staining and quantification of ZBP1 relative fluorescence intensity; (C) Western blot analysis of PANoptosis‐related proteins: Caspase‐8, Caspase‐3, NLRP3, GSDMD, GSDME, RIP3, and MLKL; CCK‐8 assay measuring SH‐SY5Y cell viability. *p < 0.05 compared with Blank (in a, c, h, k), C (in a, c, k), OGD‐Blank (in a, b, c, d, e, f), OGD‐2 h (in a, b, c, d, e, g, h, k) and OGD‐24 h (in a, e, j) groups; ^#^ *p* < 0.05compared with Blank (in a, c, d, e, g, h, i, j, k), C (in a, c, e, g, h, k), OGD‐Blank (in c), OGD‐C (in a, c, d, e, g, h, k) and OGD‐24 h (in all) groups; ^&^ *p* < 0.05 compared with Blank (in c, j, k), C (in c, e, f, j), OGD‐Blank (in a, b, c, d, e, f, g, i, j, k) and OGD‐C (in b, e, f, i, j) groups. *n* = 3.

Furthermore, western blot analysis showed that PANoptosis‐related proteins were significantly upregulated in the OGD‐Blank and OGD‐2 h groups, but markedly suppressed in the OGD‐24 h group (*p *< 0.05) (Figure 4C). CCK‐8 assays demonstrated that neuronal viability was significantly reduced in the OGD‐Blank and OGD‐2 h groups compared with the OGD‐24 h group, which exhibited significantly increased viability (*p *< 0.05) (Figure 4C).

## 4 Discussion

With an aging population, the public health burden of myocardial infarction and stroke is expected to increase due to their rising prevalence among older adults. Understanding the molecular mechanisms underlying post‐myocardial infarction stroke is critical for improving clinical management and developing effective prevention strategies [25]. Exosomes are increasingly recognized as natural carriers of noncoding RNAs (ncRNAs), mediating intercellular communication relevant to ischemic stroke and myocardial infarction [26]. In the present study, we found that exosomes isolated from the plasma of patients with AMI are readily taken up by cultured neuronal cells. Moreover, exosomes derived from human plasma after reperfusion therapy affected SH‐SY5Y cells viability and the ZBP1/PANoptosis pathway under both normoxic and hypoxic conditions.

Cell death is a major contributor to cerebral injury following ischemic stroke. It leads to cell loss, disrupts neural networks, and ultimately impairs brain function and recovery. PANoptosis is a unified form of regulated cell death that integrates apoptosis, pyroptosis, and necroptosis. It can be activated simultaneously or sequentially under specific stress conditions, such as cerebral ischemia or hypoxia [27]. Yan et al. demonstrated that PANoptosis occurs in rat and mouse models of I/R injury, confirming its involvement in ischemic brain damage [28]. The inhibition of PANoptosis will alleviate brain damage in an ischemic injury animal model [19]. In our study, pre‐reperfusion exosomes from AMI patients induced greater PANoptosis in SH‐SY5Y cells under normoxic conditions, suggesting that these exosomes may promote neuronal cell death even in the absence of stress. In contrast, exosomes collected 2 h after reperfusion induced increased PANoptosis under oxygen‐glucose deprivation (OGD). These variations in PANoptosis under OGD conditions may reflect differences in let‐7c‐5p content among exosomes collected at different time points (pre‐reperfusion, 2 h pos‐reperfusion, and 24 h post‐reperfusion).

Exosomal miRNAs have attracted significant attention for their ability to mediate intercellular communication from donor cells to distant recipient cells [24]. Let‐7c‐5p, a widely expressed and evolutionarily conserved miRNA, has been shown to confer protection against ischemic stroke [9]. Exosomal let‐7c‐5p may play a vital role in neuronal cells. Joerger‐Messerli et al. showed that the MSC‐derived extracellular vesicles (EVs) attenuated the OGD/R‐induced increase in Caspase‐3 expression through their let‐7‐5p miRNA cargo [29]. Additionally, ischemic stroke has been associated with reduced inflammation and improved prognosis through elevated exosomal let‐7 levels [30]. In our study, exosomal let‐7c‐5p levels from AMI patients were highest before reperfusion, decreased at 2 h post‐reperfusion, and partially recovered at 24 h. This finding, together with public dataset analyses showing elevated let‐7c‐5p in myocardial infarction patients [31], suggests that the initial increase in let‐7c‐5p may be part of a cardiac protective response. SH‐SY5Y cells incubated with exosomes collected 2 h post‐reperfusion under OGD/R exhibited the lowest viability and the highest PANoptosis. These results, consistent with previous studies, indicate a negative correlation between exosomal let‐7c‐5p levels and neuronal cell injury under OGD/R conditions [32]. It is well known that ZBP1, a key cytosolic DNA sensor, initiates innate immune responses [33]. Our results showed that overexpression of let‐7c‐5p downregulates ZBP1, indicating that let‐7c‐5p may target ZBP1. Therefore, exosomal let‐7c‐5p from AMI patients may affect PANoptosis through regulating ZBP1.

ZBP1 is a cytosolic nucleic acid sensor that serves as a key mediator of programmed cell death pathways. It plays a crucial role in the pathophysiology of the nervous system and is implicated in the progression of brain injury [34]. Acting as a central hub, ZBP1 connects various cell death signaling proteins to assemble the PANoptosome complex [35]. Mutoh et al. characterized the spatial and temporal expression of ZBP1 in the brain following stroke using a mouse photothrombotic model. Their results showed that ZBP1 reaches its highest transcriptional levels during the subacute phase, between 3 and 14 days after stroke induction [15]. Cui et al. demonstrated that ZBP1 acts as an initiator of PANoptosis in endothelial cells under hypoxia/reoxygenation (H/R) stress, and that its selective inhibition significantly attenuates PANoptosis‐like cell death [36]. In our study, we found that ZBP1 expression was highest in SY5Y cells incubated with exosomes collected before reperfusion therapy under normal conditions and also peaked in cells treated with exosomes collected 2 h after reperfusion therapy under OGD conditions. ZBP1 mediated PANoptosome assembly initiates pyroptosis, apoptosis, and necroptosis by activating the NLRP3/ASC/CASP1/GSDMD, CASP3/6/7, and MLKL pathways, ultimately leading to PANoptosis [37, 38]. In our current research, the expression levels of representative markers of pyroptosis (e.g., NLRP3), apoptosis (e.g., CASP3), and necroptosis (e.g., MLKL) were found to correlate with ZBP1 levels under both normal and OGD conditions. These findings suggest that plasma‐derived exosomes from AMI patients may induce neuronal injury when applied before reperfusion therapy under normal conditions or shortly after reperfusion therapy (2 h) under OGD conditions.

Plasma exosomes can cross the blood‐brain barrier and enter the central nervous system [39, 40]. The neuronal cells can internalize plasma‐derived exosomes in vitro [24, 41]. Additionally, myocardial infarction alone can induce brain injury in animal models through heart‐derived exosomal miRNAs [42]. Merkler et al. reported that patients were at an elevated risk of stroke within 3 months after AMI [4]. Similarly, Kajermo et al. found that percutaneous coronary intervention (PCI) could lower the incidence of AIS in AMI patients [43]. However, Aggarwal et al. noted that PCI, although rare, can itself trigger perioperative AIS, often occurring within 24 h post‐reperfusion therapy [44]. In our study, exosomal let‐7c‐5p levels were slightly higher at 24 h post‐reperfusion therapy compared to 2 h post‐reperfusion therapy, coinciding with reduced ZBP1 expression and decreased PANoptosis under OGD conditions. These in vitro findings suggest that the exosomal let‐7c‐5p/ZBP1/PANoptosis axis before or after reperfusion therapy may partially explain these clinical observations.

The following limitations should be acknowledged in the current study. First, we did not investigate the molecular mechanisms underlying the observation that elevated exosomal let‐7c‐5p levels are associated with increased ZBP1 expression and enhanced PANoptosis under normal conditions. Other exosomal miRNAs may also contribute to this effect. Second, blood samples were collected at only three discrete time points—before, 2 h post, and 24 h post‐reperfusion therapy. While these time points were chosen to capture the major phases of reperfusion, additional intermediate time points (e.g., within the first 48–72 h) and longer‐term follow‐up (days to months) would be needed to fully delineate the kinetics of exosomal miRNA release and its temporal relationship with neuronal PANoptosis across both acute and chronic phases. Third, the use of cultured cells may not fully recapitulate the complex in vivo interactions between the heart and brain, potentially limiting the translational relevance of our findings. Animal models (e.g., AMI mouse models with exosomal let‐7c‐5p modulation) are warranted in future studies to determine whether this axis affects PANoptosis and neurologic outcomes in a systemic context. Fourth, direct binding of let‐7c‐5p to ZBP1 mRNA has not yet been experimentally confirmed. Future studies employing luciferase reporter assays with wild‐type and mutant ZBP1 3' UTR constructs—specifically targeting the predicted binding site within this canonical regulatory region—are warranted to definitively establish ZBP1 as a direct target.

In conclusion, this findings demonstrated that exosomes derived from the plasma of AMI patients, collected at different time points (pre‐reperfusion, 2 h post‐reperfusion, and 24 h post‐reperfusion), directly influence PANoptosis levels in cultured neuronal cells under both normal and OGD conditions. These effects appear to be partially mediated by increased expression of let‐7c‐5p, which is associated with the downregulation of ZBP1. However, the impact of exosomes on ZBP1/PANoptosis was not entirely consistent with let‐7c‐5p expression levels across conditions, suggesting that other regulatory factors may also be involved. Further research is needed to elucidate the precise molecular mechanisms underlying these observations.

## Author Contributions

Conceptualization, Zhiyong Zhang., Xinyu Wang., and Zhiguang Wang. Methodology, Lei Yan., Wei Li., Yuzhi Shao., and Shaohan Zhang. Formal Analysis, Kerui Gong. Investigation, Wendian Yang. and Gang Fu. Funding acquisition, writing – review and editing, Jie Yan., Guo Shao. All authors have read and agreed to the published version of the manuscript.

## Ethics Statement

The experiment was approved by the Committee on the Ethics of The Third People's Hospital of Longgang District Shenzhen (approval number: KY‐KT‐20240024).

## Conflicts of Interest

The authors declare no conflicts of interest.

## Supporting information


Supporting File 1

## Data Availability

The data that support the findings of this study are openly available in NCBI Sequence Read Archive (SRA) repository at http://www.ncbi.nlm.nih.gov/bioproject/1326256, reference number PRJNA1326256. The microRNA sequencing datasets generated and analysed during the current study are available in the NCBI Sequence Read Archive (SRA) repository, accession number PRJNA1326256 ((http://www.ncbi.nlm.nih.gov/bioproject/1326256). (https://www.ncbi.nlm.nih.gov/sra/SRX30435715-SRX30435717)). Other data generated in the present study are included in the figures and/or tables of this article.
