# Supplementary material for: Exosomal let‐7c‐5p From AMI Patient Plasma Modulates Neuronal PANoptosis via ZBP1 In Vitro
Source: Cell Biochem Funct. 2026 Oct 8;44(10):e70318. doi: 10.1002/cbf.70318 (PMC13647479; doi:10.1002/cbf.70318)
Supplement: Supplementary file 1 [file CBF-44-e70318-s001.docx]

**Figure S1. Bioinformatic analysis of plasma exosomal contents in patients with AMI.**

(A-C) VENN diagrams of exosomal miRNA species before, 2 h after, and 24 h after reperfusion therapy. (D) Exosomal miRNA KEGG enrichment analysis. (E) Exosomal miRNA GO enrichment analysis.
